# Evaluating the outcomes of submerged co-cultivation: production of lovastatin and other secondary metabolites by *Aspergillus terreus* in fungal co-cultures

**DOI:** 10.1007/s00253-019-09874-0

**Published:** 2019-05-16

**Authors:** Tomasz Boruta, Iwona Milczarek, Marcin Bizukojc

**Affiliations:** Faculty of Process and Environmental Engineering, Department of Bioprocess Engineering, Lodz University of Technology, ul. Wolczanska 213, 90-924 Lodz, Poland

**Keywords:** *Aspergillus terreus*, Secondary metabolites, Lovastatin, Co-culture, Submerged cultivation

## Abstract

The goal of the study was to compare the production of secondary metabolites by *Aspergillus terreus* ATCC 20542 under the conditions of submerged mono- and co-cultivation. The suggested experimental scheme encompassed a diverse set of co-culture initiation strategies differing mostly with respect to the development stage of tested fungal strains at the moment of their confrontation. Three species of filamentous fungi exhibiting distinct patterns of morphological evolution under submerged conditions, namely *Penicillium rubens*, *Chaetomium globosum*, and *Mucor racemosus*, were selected as the co-cultivation partners of *A. terreus*. The choice of the co-cultivated species and the approach of co-culture triggering noticeably influenced the levels of lovastatin (mevinolinic acid), (+)-geodin, asterric acid, and butyrolactone I in the broth. Even though the evaluated co-cultures did not lead to the increased titers of lovastatin relative to standard monocultures, the biosynthesis of the remaining three metabolites was either enhanced or inhibited depending on the experimental variant. The production of butyrolactone I turned out to be particularly affected by the presence of *C. globosum*. Interestingly, in the *A. terreus*/*C. globosum* co-cultures, the decrease of lovastatin concentration was recorded. According to the most probable scenario, lovastatin was in this case converted to monacolin J acid, a polyketide molecule that may be applied as a substrate for the synthesis of statin drugs. The study revealed that the spores of two distinct fungal species, namely *A. terreus* and *C. globosum*, co-agglomerate under submerged conditions to form pellets. Finally, the biosynthetic performance of co-cultures involving four fungal species was evaluated.

**Electronic supplementary material:**

The online version of this article (10.1007/s00253-019-09874-0) contains supplementary material, which is available to authorized users.

## Introduction

Fungal secondary metabolites constitute a rich group of bioactive molecules, some of which are considered to be promising leads in drug development projects (Adrio and Demain 2003; Brakhage 2013; Demain 2014; Keller et al. 2005). While it has been 90 years since an antibiotic penicillin was serendipitously discovered as a metabolic product of a mold *Penicillium rubens* (originally referred to as *Penicillium notatum*) (Fleming 1929; Houbraken et al. 2011), the scientific and industrial interest in fungal secondary metabolism remains unabated. Notably, the strategies aimed at elucidating the complex machinery of biosynthetic pathways and inducing the formation of previously unknown chemical structures are becoming more and more sophisticated. Since the production of secondary metabolites is typically associated with specific environmental cues, mimicking the signals encountered under natural conditions is an effective strategy to influence the secondary metabolic pathways, i.e., the ones that are believed to be unnecessary for cellular growth and energy processes but provide a competitive ecological advantage for the producing strain. A diverse set of experimental approaches aimed at deciphering the biochemical potential of filamentous fungi encompasses varying medium compositions and growth conditions, isolation, and cultivation of fungal strains under extreme conditions, introducing genetic modifications and performing co-cultivation that simulates the naturally occurring microbial interactions (Bode et al. 2002; Brakhage and Schroeckh 2011; Chávez et al. 2015; Marmann et al. 2014; Netzker et al. 2015). Employing a microbial co-culture, defined by Bader et al. (2010) as “anaerobic or aerobic incubation of different specified microbial strains under aseptic conditions,” gains attention not only in the context of secondary metabolism. Most importantly, the co-culture approach opens the door for understanding the dependencies between the members of microbial populations found in the gut, in fermented food products or, more recently, in synthetic microbial consortia (McCarty and Ledesma-Amaro 2019; Stadie et al. 2013; Tramontano et al. 2018). The expansion of the research scope from mono- to co-cultivation is frequently observed in the recent scientific endeavors. Notably, the production of secondary metabolites in co-cultures is more frequently addressed qualitatively by employing agar media and focusing on the “on/off” biosynthetic scenarios yielding novel and potentially useful molecules (Bertrand et al. 2014). In the context of liquid media–based cultivation, one may anticipate that substituting the conventional monoculture runs with co-culture variants would ultimately lead to the elevated or lowered levels of secondary metabolites in the broth. For example, Chatterjee et al. (2016) performed the experiments with the use of non-agitated liquid media and noted the decrease of fumonisin B1, B2, and B3 levels in the dual cultures of *Fusarium verticillioides* with *Clonostachys rosea* compared with the monocultures of *F. verticillioides*. Importantly, the approach of submerged fungal co-cultivation aimed at the production of secondary metabolites remains rather unexplored and still requires thorough quantitative investigation, preferably in the context of the industrially relevant organisms.

The present study was focused on *Aspergillus terreus*, a filamentous fungus used for the industrial production of lovastatin, also known as mevinolin, a cholesterol-lowering drug. Lovastatin, like other statins, blocks cholesterol biosynthesis via competitive inhibition of 3-hydroxy-3-methylglutaryl-CoA reductase, an enzyme involved in the mevalonate pathway. It is biosynthesized as the β-hydroxy acid, often referred to as mevinolinic acid, and then transformed into its corresponding lactone form during the downstream processing steps. Next to a penicillin-producing fungus *Penicillium rubens*, *A. terreus* is regarded as a textbook fungal workhorse employed in the industrial production of secondary metabolites. In addition, it is widely used in itaconic acid manufacturing (Okabe et al. 2009). In the present work, the strain *A. terreus* ATCC 20542 was chosen as the research subject. This microorganism is primarily recognized as a parent of high-yielding industrial strains employed in the industrial lovastatin production (Mulder et al. 2015). Furthermore, it has been studied extensively in the conventional liquid monocultures, both in shake flasks and bioreactors (e.g., Bizukojc and Ledakowicz 2010; Boruta and Bizukojć 2016; Casas López et al. 2003). In the context of the present work, it should be noted that developing an essential analytical toolbox and gathering cultivation-related experience while conducting the conventional monocultures of *A. terreus* ATCC 20542 greatly facilitated the subsequent experimental efforts, namely the design, performance, and evaluation of co-culture runs. It has been established in our previous work that *A. terreus* ATCC 20542 is capable of producing a number of secondary metabolites in liquid media, including lovastatin (as the main product), (+)-geodin, asterric acid, and butyrolactone I (Boruta and Bizukojć 2016). In fact, the latter three molecules can be viewed as the major metabolic by-products of lovastatin biosynthesis. In the chemical manufacturing perspective, they comprise a set of unwanted chemical species that need to be removed during the isolation and purification steps. In order to account for the main by-products of the process and expand the analytical scope of the study, the present work focused not only on the comparison of lovastatin (mevinolinic acid) biosynthesis by *A. terreus* ATCC 20542 in mono-and co-cultures but also on the quantitative determination of abovementioned three secondary metabolites co-produced with the target compound.

In the previous work of Chen et al. (2015), two new butyrolactone derivatives were discovered as a result of co-cultivation of *A. terreus* with *Bacillus subtilis* and *Bacillus cereus* on the solid rice medium. Moreover, the authors recorded up to 34-fold increase in the accumulation of several metabolites in co-cultures compared with monocultures. Importantly, the study provided the direct evidence that the biosynthetic repertoire of *A. terreus* can be expanded via co-cultivation approach. The metabolic capabilities of the employed *A. terreus* strain (previously isolated from the lake sediments) were, however, evaluated solely in the context of solid media cultivation. Furthermore, lovastatin was not detected in the tested samples (Chen et al. 2015). In a different study, Losada et al. (2009) investigated the effects of the competition on the production of secondary metabolites by a number of *Aspergillus* species, including *A. terreus*. Interestingly, the authors concluded that *A. terreus* NIH2426, a clinical strain with the sequenced genome, competes poorly with other *Aspergilli* and is typically outgrown by the competitors in co-cultures. The experiments were conducted with the use of solid agar media and did not include submerged runs. Moreover, similarly as in the aforementioned report of Chen et al. (2015), lovastatin formation was not addressed. To the best of our knowledge, the production of lovastatin and other secondary metabolites by *A. terreus* in the submerged co-cultures has never been evaluated in literature. Until now, it has not been established whether the submerged liquid co-cultivation can be considered as a justified and valuable approach in the context of intensifying secondary metabolites production by *A. terreus*. The primary motivation behind the present study was to address this issue.

The goal of this work was to compare the levels of secondary metabolites produced by *A. terreus* ATCC 20542 in the mono- and co-cultures under submerged conditions. Several strategies of fungal co-culture initiation were suggested to systematically evaluate the biosynthetic capabilities of the investigated strain.

## Materials and methods

### Experimental approach

**Fig. 1:**
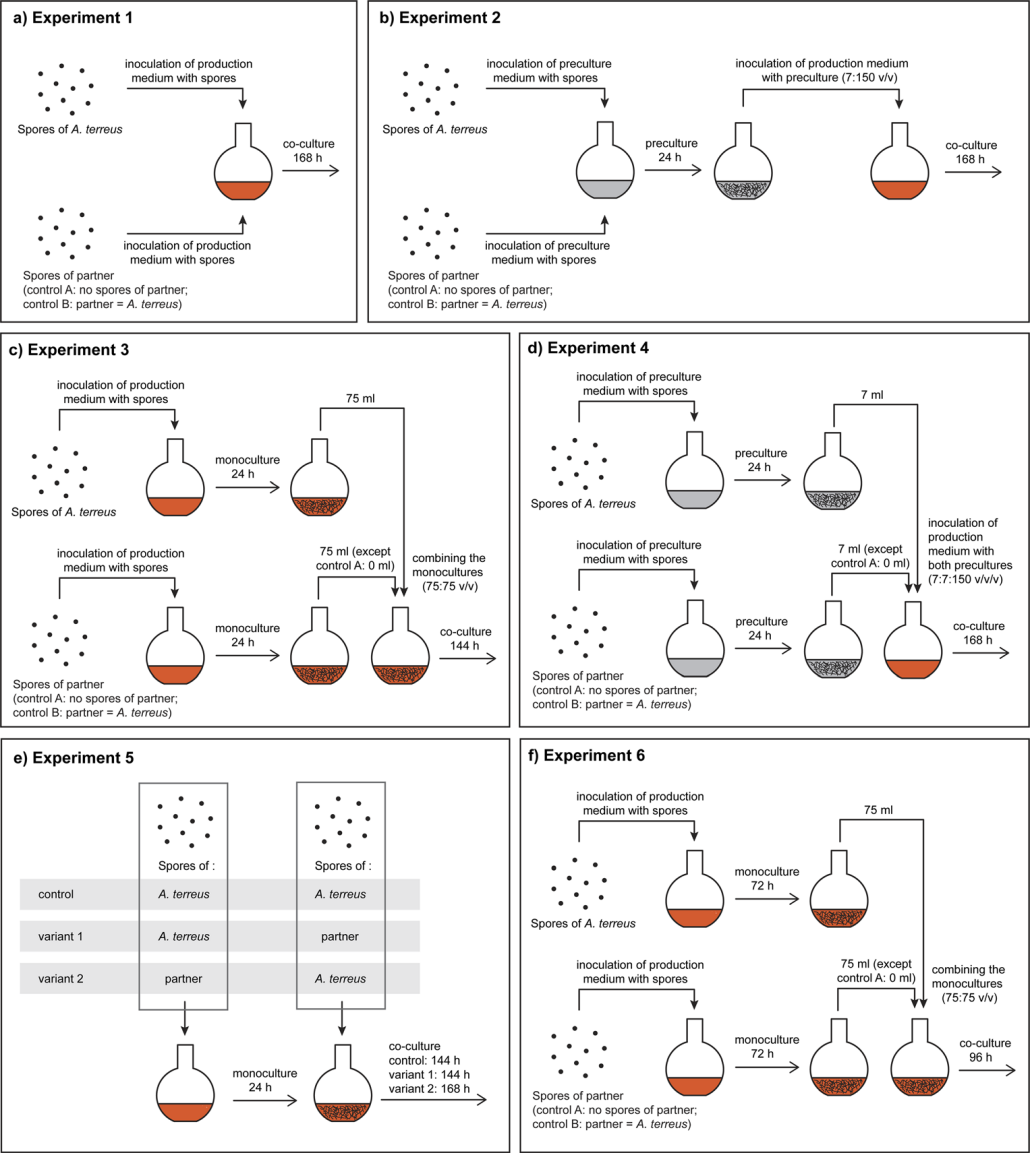
Scheme depicting the co-culture initiation types applied in the present study. The suggested strategies, depicted respectively as “Experiments 1–6”, were as follows: the confrontation of spores of two fungal species without (**a**) and with (**b**) the use of preculture medium, the contact of fungal mycelia after 24 h of separate growth of two fungal species without (**c**) and with (**d**) the use of preculture media, the confrontation of spores of *A. terreus* with the 24-h mycelium of the other species (and vice versa) (**e**), and the contact of 72-h mycelia of both involved species (**f**). The scheme includes the experimental controls being the monocultures of *A. terreus*

The co-cultures were started in accordance with six different approaches, which are schematically depicted in Fig. 1 as “Experiments 1–6.” In this diverse set, most of the proposed designs assumed “equal chances” with respect to the development stage and age of the contacted strains, i.e., spores of *A. terreus* versus spores of the fungal partner, 24-h mycelium versus 24-h mycelium or 72-h mycelium versus 72-h mycelium (Fig. 1). An exception was the confrontation between the spores of *A. terreus* and 24-h mycelium of the partner (and vice versa). Regarding the duration of co-cultivation runs, the following approach was suggested: each co-culture would last 168 h starting from the moment of introducing *A. terreus* into the production medium. This explains why the co-cultivation was continued for 168 (Fig. 1a, b, d, e), 144 (Fig. 1c, e), or 96 h (Fig. 1f) depending on the experiment.

In the liquid co-culture, the levels of *A. terreus* metabolites were expected to be influenced both by the presence of a specific co-culture partner and by the quantity of fungal cells present in the medium. To provide a comparative perspective, the conducted experiments included the *A. terreus* control variants (monocultures) that differed in terms of volumes, spore numbers, or inoculum loads (Fig. 1). The goal was to compare the effects of quantitative bioprocess–related manipulations involving exclusively *A. terreus* (grown in monocultures) with the outcomes of confrontations with different fungal strains.

The fungi selected for the study were initially planned to be co-cultivated in pairs, i.e., *A. terreus* and a partner strain, according to the exact scheme presented in Fig. 1. However, an additional “all-in-one” experiment involving all four tested strains was also conducted. It needs to be mentioned here that the metabolic activities of the co-culture partners of *A. terreus* were not under consideration in the current study.

### Strains

The selection of strains serving as co-culture members was mostly based on our previous experience with morphological studies of various fungal species (Kowalska et al. 2018). Three strains of filamentous fungi, namely *Penicillium rubens* ATCC 9178, *Chaetomium globosum* ATCC 6205, and *Mucor racemosus* ATCC 7924, were chosen as the co-cultivation partners of *A. terreus*. The rationale behind this decision was to open the door for a wide spectrum of interactions occurring between fungi displaying significant diversity of growth characteristics and morphological development under submerged conditions (for details, see Kowalska et al. 2018).

### Inoculation

Inoculation was carried out by transferring fungal spores from the test tubes with agar slants into the flasks containing sterile liquid media. The portion of liquid medium (4 ml) was transferred from a flask into a tube and then a sterile disposable pipette was used to prepare a suspension of spores, which was then transferred into a flask to obtain 10^9^ spores per liter of medium.

### Cultivation conditions

All cultivation runs described in the present report (both in mono- and co-culture mode) were performed at 28 °C in a shaking incubator Certomat® BS-1 (B. Braun Biotech International, Germany) with the use of flat-bottomed shake flasks (working volume 150 ml, total volume 500 ml). The rotary speed was set to the value of 110 rpm (rotations per minute) (Kowalska et al. 2018).

### Medium

Since the study was focused on *A. terreus* and its biosynthetic performance, the medium composition effective in the context of lovastatin production was selected (Bizukojc and Ledakowicz 2007; Casas López et al. 2003): lactose, 20 g l^−1^; yeast extract, 4 g l^−1^; KH_2_PO_4_, 1.51 g l^−1^; NaCl, 0.4 g l^−1^; MgSO_4_·7H_2_O, 0.51 g l^−1^; and biotin, 0.04 mg l^−1^. The trace elements solution was also added to the medium (1 ml per 1 l of medium). The composition of the trace elements solution was as follows: ZnSO_4_·7H_2_O, 1 g l^−1^; Fe(NO_3_)_3_·9H_2_O, 2 g l^−1^; MnSO_4_, 50 mg l^−1^; Na_2_B_4_O_7_·10H_2_O, 100 mg l^−1^; Na_2_MoO_4_·2H_2_O, 50 mg l^−1^; and CuSO_4_·5H_2_O, 250 mg l^−1^ (Bizukojc and Ledakowicz 2007). The pH of the medium was adjusted to 6.5 by using NaOH solution. The medium was sterilized by autoclaving at 121 °C for 20 min.

### Chemical analysis

After the cultivation run, the biomass was separated by filtration and discarded, whereas the filtrate was stored at − 20 °C. All analyses were performed with the use of an ultra-performance liquid chromatography system Acquity (Waters, USA) coupled with high resolution mass spectrometer Synapt G2 (Waters, USA). The concentrations of lovastatin (in its β-hydroxy acid form), (+)-geodin, asterric acid, and butyrolactone I in the liquid samples were determined according to the procedure described previously by Boruta and Bizukojć (2016). The semi-quantitative analysis of a molecule putatively identified as monacolin J acid was performed with the use of TargetLynx™ software (Waters, USA) on the basis of peak area values corresponding to the fragment ion of *m*/*z* = 303.1969, which exhibited the greatest intensity on the mass spectrum.

### Microscopic analysis

Microscopic images were collected by using the Olympus BX53 light microscope and analyzed with the aid of Olympus cellSens Dimension Desktop 1.16 software (Olympus Corporation, Japan). The details of the microscopic procedure can be found in the previous report of Kowalska et al. (2018).

## Results

The outcomes of submerged co-cultivation of *A. terreus* with *P. rubens*, *C. globosum*, and *M. racemosus* were evaluated according to six experimental schemes depicted as “Experiments 1–6” in Fig. 1 and the results are presented below.

### Experiments 1–4: “spores/spores” and “24-h mycelium/24-h mycelium” contact of two species

**Fig. 2:**
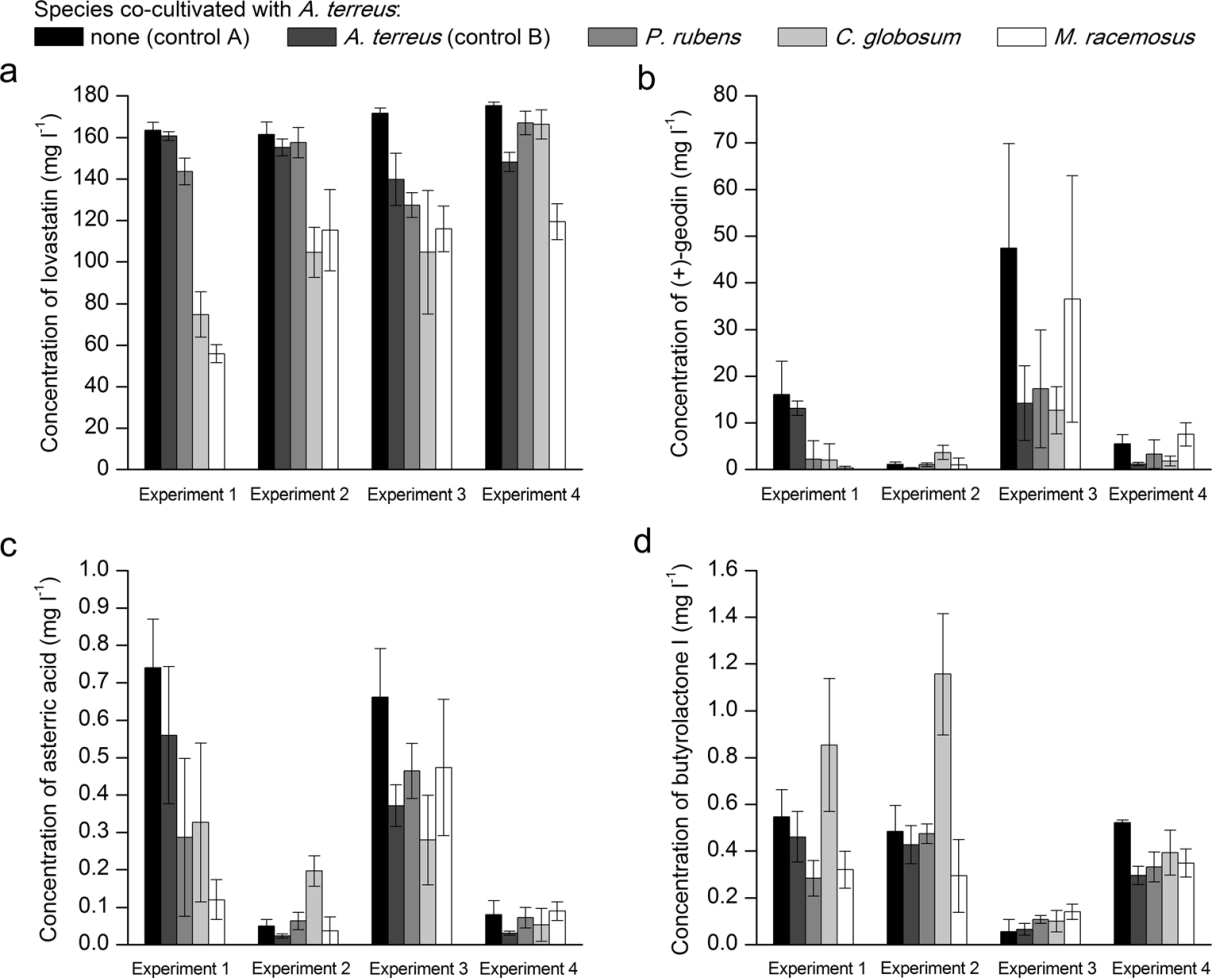
Final concentration of lovastatin (mevinolinic acid) (**a**), (+)-geodin (**b**), asterric acid (**c**), and butyrolactone I (**d**) in submerged mono- and co-cultures involving *A. terreus*. The results correspond to “Experiments 1–4”, i.e., the co-culture initiation based on the “spores/spores” and “24-h mycelium/24-h mycelium” contact of two fungal species. The species co-cultivated with *A. terreus* (*P. rubens*, *C. globosum*, or *M. racemosus*) are indicated. Error bars represent standard deviation values determined on the basis of experimental triplicates

The first set of results regarding the levels of lovastatin, (+)-geodin, asterric acid, and butyrolactone I (Fig. 2) corresponds to the co-cultures started by contacting the spores or 24-h mycelia of two fungal species, as schematically illustrated in Fig. 1a, b, c, and d as Experiments “1,” “2,” “3,” and “4,” respectively. The control runs were included in the experimental set to reflect the consequences of varying the load of *A. terreus* spores or the monoculture volume. Specifically, in the “spores/spores” variants (Fig. 1a, b), “control A” corresponded to a standard load of spores transferred from an agar slope to liquid medium (10^9^ spores per liter), whereas in “control B,” the number of spores was doubled (2 × 10^9^ spores per liter). The co-cultivation runs were performed according to two distinct medium composition-related strategies, namely without (Fig. 1a and c) and with (Fig. 1b and d) the use of preculture medium.

Considering the triplicate-based results obtained in this part of the study, the standard mono-culture (control A) turned out to be the most effective approach in terms of stimulating lovastatin production, whereas the lowest level of this metabolite was recorded for the “spores/spores” co-culture involving *M. racemosus* (Fig. 2a). Interestingly, when either *P. rubens* or *M. racemosus* were confronted with *A. terreus* after 24 h of growth in the preculture medium, the final titers of lovastatin exceeded the concentration observed in “control B,” but not in “control A” (Fig. 2a). Generally, the mean lovastatin titers reached in the “control A” flasks were noticeably closer to the values recorded for *P. rubens* co-cultures than for *C. globosum* or *M. racemosus* variants (Fig. 2a).

The concentration values corresponding to (+)-geodin (Fig. 2b) and asterric acid (Fig. 2c) were relatively more varied than those of lovastatin and this behavior was reflected by the error bars representing the standard deviation values based on experimental triplicates (Fig. 2b, c). If a complete dataset including the results for all initiation strategies is considered, the highest levels of (+)-geodin (Fig. 2b) and asterric acid (Fig. 2c) were recorded for the “control A” variants, similarly as for lovastatin. This particular observation was not made in the case of butyrolactone I, for which the concentration values peaked in the “spores/spores” co-cultures with *C. globosum*, especially in the preculture-including variant (Fig. 2d).

### Experiment 5: “24-h mycelium/spores” contact of two species

**Fig. 3:**
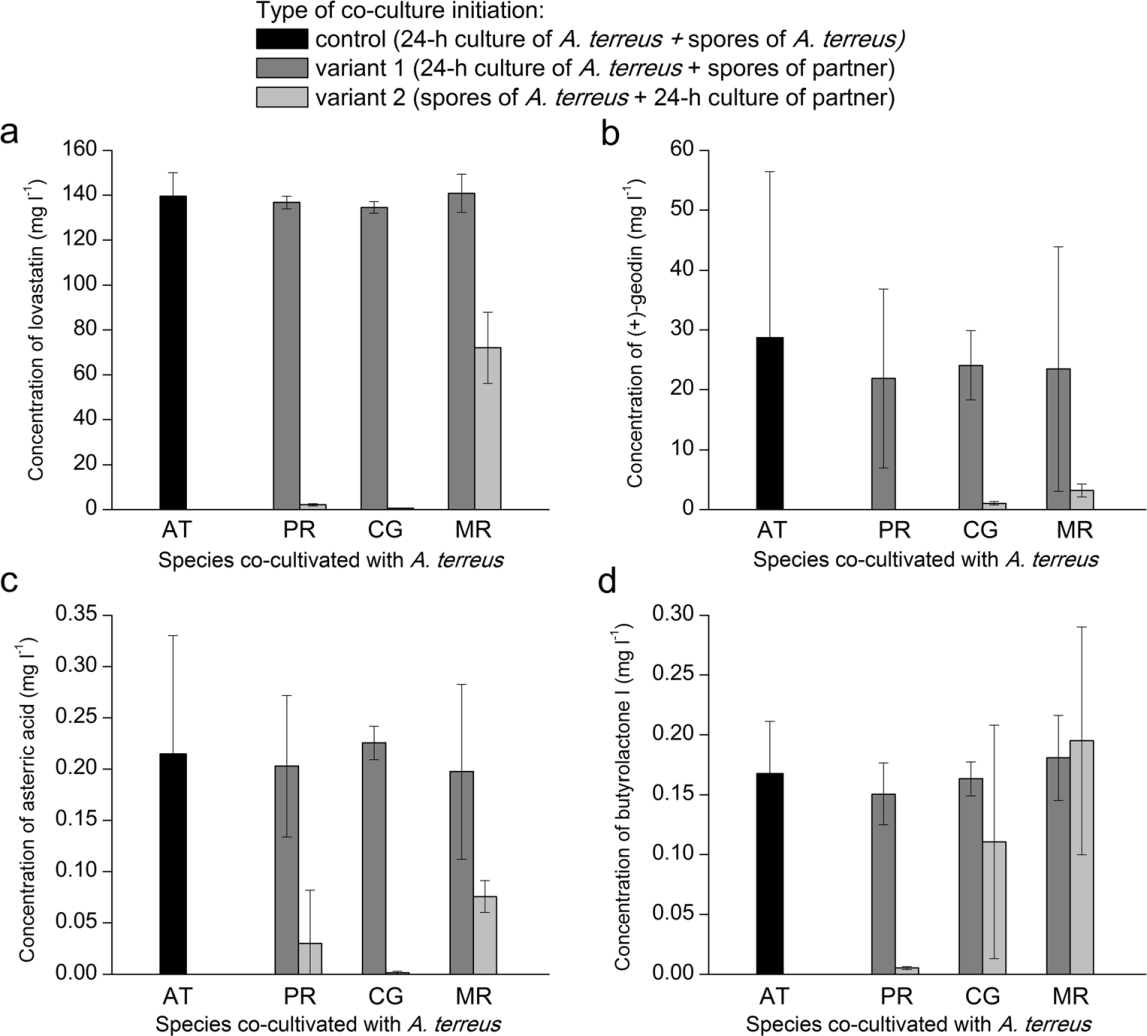
Final concentration of lovastatin (mevinolinic acid) (**a**), (+)-geodin (**b**), asterric acid (**c**), and butyrolactone I (**d**) in submerged mono- and co-cultures involving *A. terreus*. The presented results correspond to “Experiment 5”, i.e., the co-culture initiation based on the confrontation of the 24-h mycelium of *A. terreus* with the spores of the other species (variant 1) and vice versa (variant 2). The experimental control corresponds to the monoculture of *A. terreus*, where the additional load of spores was introduced to the broth after 24 h of growth. The species co-cultivated with *A. terreus* (*P. rubens*, *C. globosum*, or *M. racemosus*) are indicated. Error bars represent standard deviation values determined on the basis of experimental triplicates. AT, *A. terreus*; PR, *P. rubens*; CG, *C. globosum*, MR, *M. racemosus*

In order to investigate the “unequal chances” scenarios of the co-cultivation, one species was first allowed to grow for 24 h and then confronted with the spores of the second species, as schematically illustrated as “Experiment 5” in Fig. 1e. The main idea was to examine whether introducing the spores of *P. rubens*, *C. globosum*, or *M. racemosus* to a 24-h culture of *A. terreus* could noticeably affect the production of secondary metabolites (“variant 1” in Fig. 1e) and if the mycelium of *A. terreus* would develop and display productivity in the liquid medium already inhabited by a different fungus (“variant 2” in Fig. 1e). In the experimental control, both the spores and the 24-h culture were of the same species, namely *A. terreus* (Fig. 1e). The results of the experiment are presented in Fig. 3.

Generally, adding the spores of *P. rubens*, *C. globosum*, of *M. racemosus* to the 24-h submerged cultures of *A. terreus* did not lead to the markedly altered levels of secondary metabolites relative to the control (see “variant 1” in Fig. 3). Thus, the disturbance of the underlying biosynthetic pathways as a result of the “invasion” of foreign spores was not recorded in this case. In the reverse scenario, however, introducing the spores of *A. terreus* into the 24-h fungal cultures noticeably affected the metabolic outcomes relative to the control values (see “variant 2” in Fig. 3). With the exception of butyloactone I biosynthesis in the presence of *M. racemosus* (Fig. 3d), the concentration values recorded for this variant were lower than the levels observed in “variant 1” runs and in the controls (Fig. 3). It could be noticed that *M. racemosus* was less inhibitory in terms of lovastatin, (+)-geodin, asteric acid, and butyrolactone I formation in the *P. rubens* and *C. globosum* (Fig. 3).

### Experiment 6: “72-h mycelium/72-h mycelium” contact of two species

The chemical composition of the broth gradually changes over the course of the batch cultivation, mainly as a result of metabolic activities of the strain. It is now well-known that for some secondary metabolites, the awakening of the underlying biosynthetic pathway occurs when the growth processes slow down and the culture enters the so-called idiophase (the production phase). In turn, the presence of secondary metabolites in the medium may have an impact on the outcomes of co-cultivation. To investigate this scenario, the co-cultures initiated after 72 h of monoculture growth were evaluated (according to the scheme in Fig. 1f depicted as “Experiment 6”). In contrast to the “24-h mycelium/24-h mycelium” type of contact discussed above, the confrontation between the 72-h monocultures was associated with the presence of detectable amounts of secondary metabolites at the co-culture start. In order to monitor the increase or decrease of metabolite levels in the co-cultures, the concentration values of lovastatin, (+)-geodin, asterric acid, and butyrolactone I were determined not only at the very end of the experiment but also after 72 h of *A. terreus* monocultivation, just before the initiation of co-cultures. By taking the dilution into account, one could evaluate the accumulation of secondary metabolites in the co-culture variants during the subsequent 96 h of the run (Fig. 1f).

**Fig. 4:**
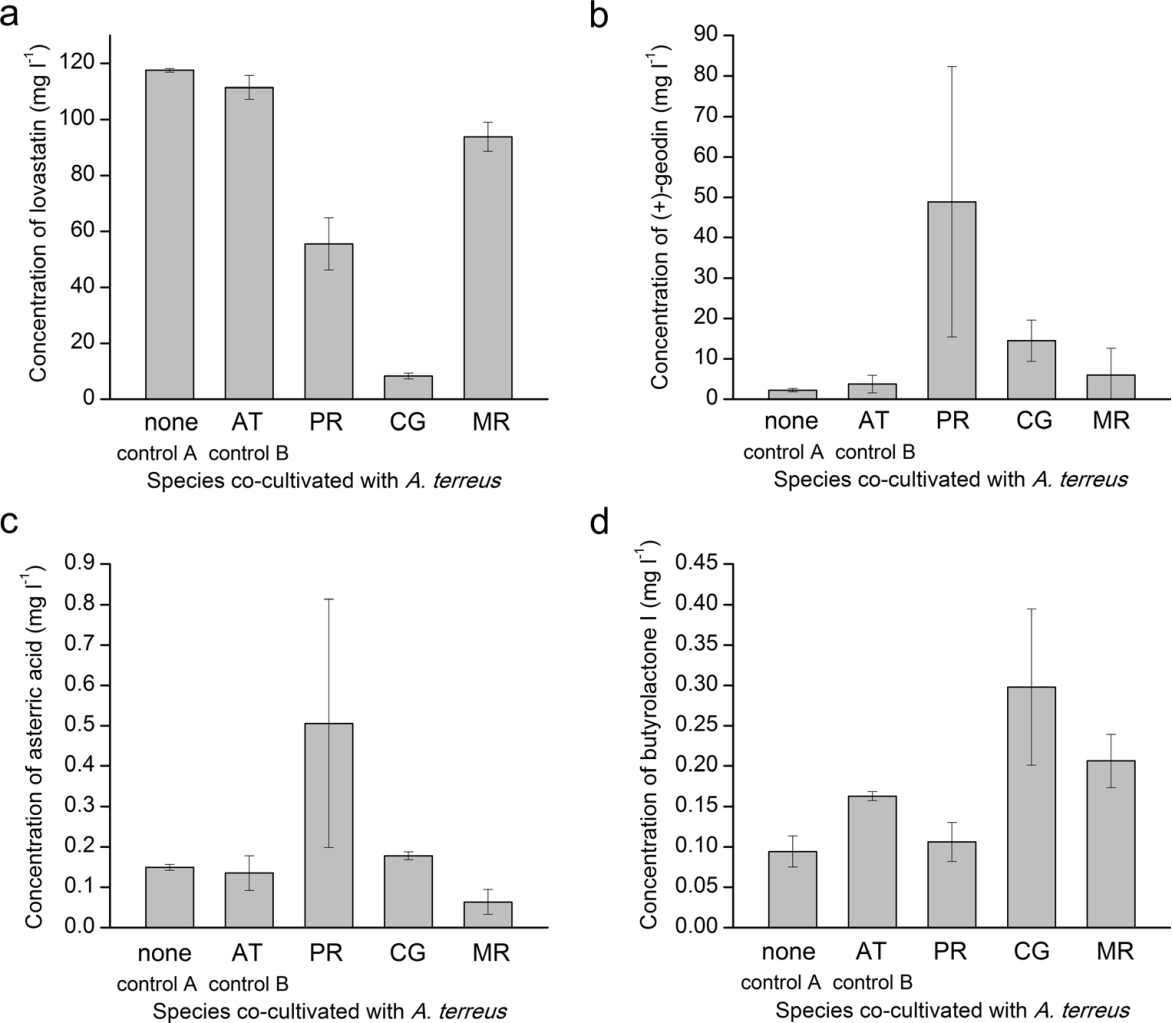
Final concentration of lovastatin (mevinolinic acid) (**a**), (+)-geodin (**b**), asterric acid (**c**), and butyrolactone I (**d**) in submerged mono- and co-cultures involving *A. terreus*. The results correspond to “Experiment 6”, i.e., the co-culture initiation based on the 72-h mycelium/72-h mycelium contact of two fungal species. The species co-cultivated with *A. terreus* (*P. rubens*, *C. globosum*, or *M. racemosus*) are indicated. Error bars represent standard deviation values determined on the basis of experimental triplicates. AT, *A. terreus*; PR, *P. rubens*; CG, *C. globosum*, MR, *M. racemosus*

According to the analysis performed at 72 h of the experiment, the concentration of lovastatin in *A. terreus* monocultures reached 80.2 ± 8.3 mg l^−1^ just before initiating the co-cultures. Since the monocultures were always combined in the ratio of 75:75 ml (i.e., the 1:2 dilution of each culture was carried out), the starting concentration in the two-species co-cultivation variants was thus estimated at the mean level of 40.1 mg l^−1^, while in the controls, the monocultivation proceeded without dilution (Fig. 1f). One could have expected the concentration of secondary metabolites in the broth to increase during the following 4 days of the experiments; however, it was not always the case (Fig. 4a). In fact, it turned out that the concentration of lovastatin in the *A. terreus*/*C. globosum* variant was visibly lower at the end of the run (i.e., after 4 days of co-culture) than at the time when the co-cultures were actually started. So, not only did the accumulation of lovastatin not occur in this case but the metabolite was degraded in the medium down to the level of 8.3 ± 1.1 mg l^−1^ (Fig. 4a), which was possibly due to the activity of enzymes secreted by *C. globosum*.

As far as the biosynthesis of (+)-geodin was concerned, the presence of this metabolite was not detected at 72 h of the run. Later on, the production of (+)-geodin did occur in all tested co-cultures and the controls, albeit the levels were clearly dependent on the species that accompanied *A. terreus* (Fig. 4b). The highest final mean concentration (48.9 mg l^−1^) was recorded for the *A. terreus*/*P. rubens* set, with the relatively large standard deviation value calculated from the triplicates (Fig. 4b). Notably, all the two-species variants displayed higher (+)-geodin levels than the controls (Fig. 4b). The outcomes with respect to asterric acid production in the “72-h mycelium/72-h mycelium” experiment were similar to the ones recorded for (+)-geodin, namely the highest mean levels of asterric acid were noted for the *A. terreus*/*P. rubens* co-culture (Fig. 4c). Moreover, asterric acid was still undetectable at 72 h of the run. As (+)-geodin and asterric acid both originate from the octaketide pathway and share the common precursors, this similarity is understandable. Nevertheless, unlike in the case of (+)-geodin, the mean titer of asterric acid in the *A. terreus*/*M. racemosus* variant was lower than the value recorded for the controls (Fig. 4c).

In the “72-h mycelium/72-h mycelium” experiment, the highest mean concentration of butyrolactone I, equal to 0.30 mg l^−1^, was recorded for the *A. terreus*/*C. globosum* co-culture (Fig. 4d). It was also noted that by confronting the 72-h biomass of *A. terreus* and *M. racemosus*, one could elevate the levels of butyrolactone I to exceed the values reached in the monoculture controls (Fig. 4d).

### Co-cultivation of four species

**Fig. 5:**
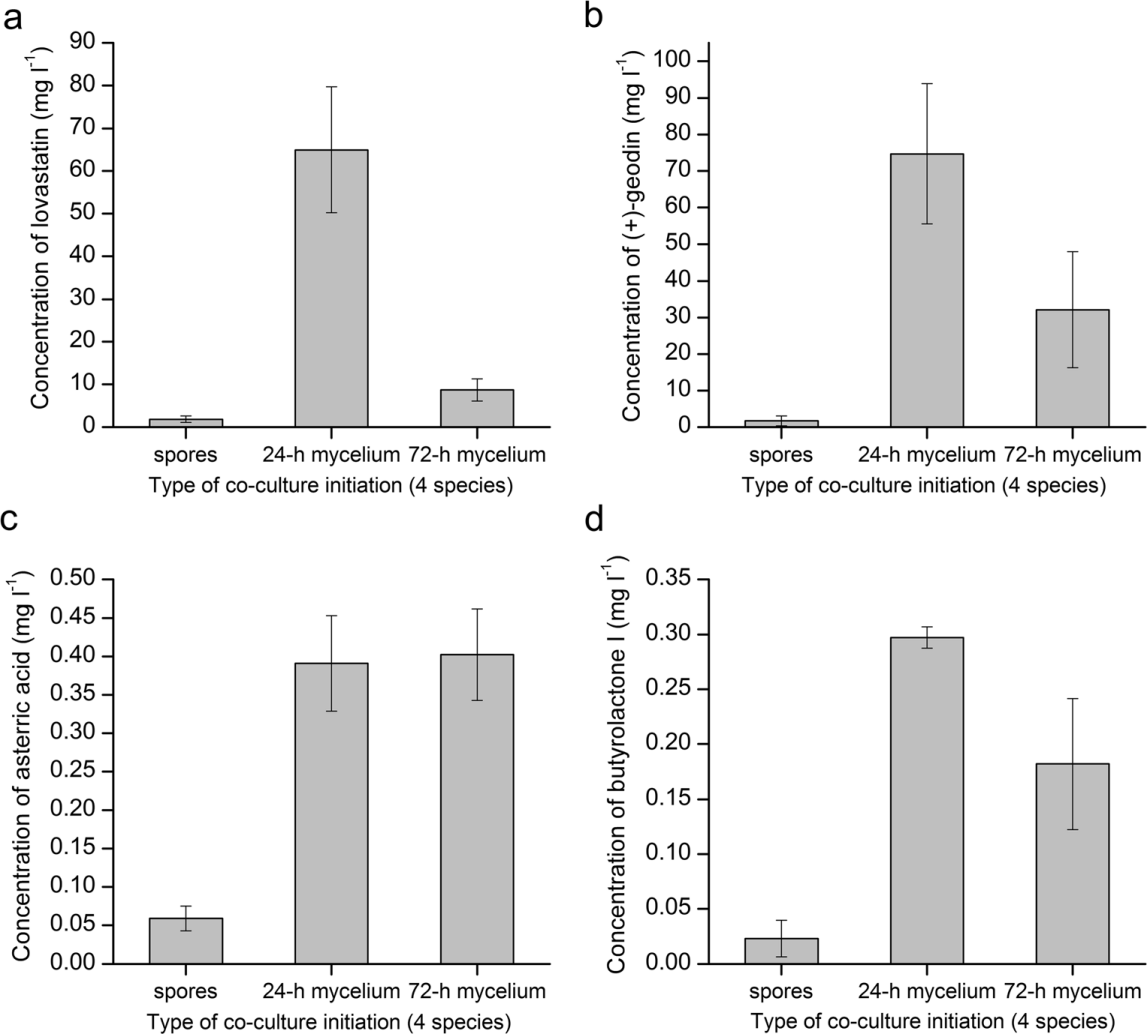
Final concentration of lovastatin (mevinolinic acid) (**a**), (+)-geodin (**b**), asterric acid (**c**), and butyrolactone I (**d**) in submerged co-cultures involving *A. terreus*, *P. rubens*, *C. globosum*, and *M. racemosus*. The types of co-culture initiation were based on the confrontation of spores, 24-h or 72-h mycelia of all four involved fungal species. Error bars represent standard deviation values determined on the basis of experimental triplicates

As an additional element of the study, three submerged co-cultures involving all four tested fungal species were evaluated. In analogy to the above-discussed cases concerning the contact of only two species, the four-species variants were initiated at the stage of fungal spores or by combining the liquid monocultures after 24 or 72 h of cultivation in shake flasks. In the case of lovastatin, (+)-geodin, and butyrolactone I production, the performance of the co-cultures started by combining the 24-h biomass was visibly better than for the two remaining approaches (Fig. 5a, b, d), whereas the mean concentration of asterric acid was comparable in the “24-h” and “72-h” contact variants (Fig. 5c). It should be mentioned that the co-culture initiated at 72 h resulted in the decrease of lovastatin concentration from 20.06 (at 72 h) to 8.72 mg l^−1^ (at 168 h) (Fig. 5a), probably as a result of chemical transformation.

Additionally, it was tempting to examine whether the four-species approach would yield novel metabolites resulting from combined enzymatic activities of the fungal co-culture members. However, the comparison of total ion chromatograms corresponding to investigated mono- and co-cultures did not reveal any previously unknown metabolites (data not shown).

It should be mentioned that in the present work, the monoculture controls were used not only for *A. terreus*, but also for *P. rubens*, *C. globosum*, and *M. racemosus* to have a clear data for studying the effects of co-cultivations. The examples of total ion chromatograms obtained for monoculture controls are provided in the supplementary material (Fig. S1). As expected, no traces of the metabolites that were analyzed in the current work were detected in the monocultures of *P. rubens*, *C. globosum*, and *M. racemosus*.

### Probable fate of lovastatin in the *A. terreus*/*C. globosum* co-cultures

**Fig. 6:**
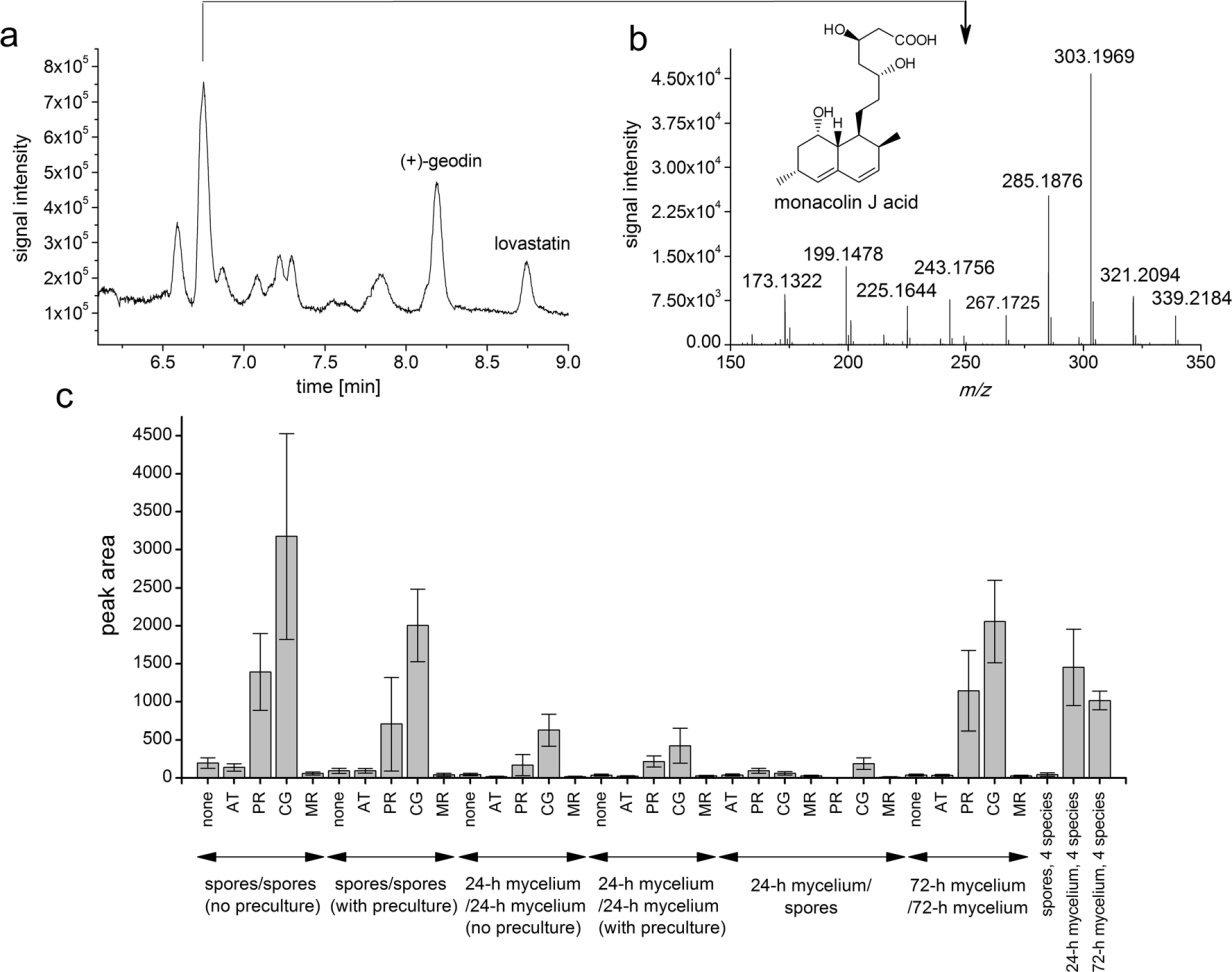
**a** Total ion chromatogram corresponding to the final day of the co-culture initiated by contacting the 72-h mycelia of *A. terreus* and *C. globosum*. The peak of the greatest area in the chromatogram is indicated by an arrow. The peaks of lovastatin (mevinolinic acid) and (+)-geodin are also depicted. **b** Mass spectrum of a molecule putatively identified as monacolin J acid. **c** Peak area values corresponding to monacolin J acid obtained over the course of the study. The structural formula of monacolin J acid is also presented in the figure. The species co-cultivated with *A. terreus* are indicated on the axis. All experimental variants described in the present report are represented on the graph. Error bars represent standard deviation values determined on the basis of experimental triplicates. AT, *A. terreus*; PR, *P. rubens*; CG, *C. globosum*, MR, *M. racemosus*

As mentioned above, the concentration of lovastatin in the run initiated by combining the 72-h monocultures of *A. terreus* and *C. globosum* decreased observably over the course of the co-culture, but the reason for this behavior was unknown. Therefore, the available literature was consulted in order to suggest the possible explanation. It turned out that the hydrolysis of lovastatin, leading to a metabolite known as monacolin J, was reported in the earlier studies. It was first observed by Komagata et al. (1986) in the cultures of the fungus *Emericella unguis*. Then, Schimmel et al. (1997) and Chen et al. (2006) focused on the production, purification, and characterization of lovastatin esterase, an enzyme responsible for this reaction, using the fungus *Clonostachys compactiuscula* as a source microorganism. In the light of these findings, the total ion chromatograms obtained in the present study for the *A. terreus*/*C. globosum* variants were carefully analyzed in order to verify the predictions regarding the presence of monacolin J in the broth. Since the reference standard of monacolin J was not available, the putative identification was based on literature data, taking into account both the recorded *m*/*z* values and the fragmentation patterns (Li et al. 2017). Not only was the mass spectrum of monacolin J (in its β-hydroxy acid form, often referred to as “monacolin J acid” in literature) detected for the *A. terreus*/*C. globosum* variants of the “72-h mycelium contact” experiment, but it corresponded to the most dominant peaks on the total ion chromatograms (Fig. 6). Therefore, the hydrolysis of lovastatin was probably responsible for the decrease of its concentration levels in this case. The question remained whether *C. globosum* is a species generally capable of hydrolyzing lovastatin during the co-cultivation with *A. terreus* or perhaps the co-culture based on “72-h mycelium” contact of the two species could have been regarded as exceptional in this respect. To address this issue, the peak areas corresponding to monacolin J acid were extracted from all the available total ion chromatograms collected over the course of this study to provide the semi-quantitative and relative evaluation regarding the formation of this metabolite in different experimental variants. Interestingly, for all the suggested co-culture initiation strategies, the peak areas attributed to monacolin J acid were greater for *A. terreus*/*C. globosum* co-cultures than for all other examined fungal pairs (Fig. 6c), with the highest mean value recorded for the “spores/spores, without preculture” experiment (Fig. 6c). Notably, in the case of the co-cultivation of four species, the presence of monacolin J acid was evident only when the 24-h or 72-h mycelia were combined, whereas the confrontation of spores was not effective in this context (Fig. 6c).

## Discussion

The study concerned the submerged co-cultivation of *Aspergillus terreus* with other filamentous fungi, the topic that has not been yet investigated. Therefore, it was not possible to provide the comparison of the results obtained here with the previously published data. Nevertheless, the key aspects of the present work and the main differences between the mono- and co-cultivation approaches will be discussed below.

The decrease of lovastatin concentration observed in the current study, particularly in the case of the *A. terreus*/*C. globosum* co-cultures, was probably due to the enzymatic hydrolysis leading to monacolin J acid. It should be mentioned here that there are two possible sources of monacolin J acid in the broth, namely the abovementioned hydrolytic reaction and, since this metabolite is a precursor of lovastatin in the biosynthetic pathway, the direct secretion by *A. terreus* cells. This explains why one could find the traces of monacolin J acid not only as a result of co-cultivation, but also in the *A. terreus* monocultures (Fig. 6c). The origin of monacolin J acid in the *A. terreus*/*P. rubens* variants, where the areas of the peaks attributed to monacolin J acid were relatively high, remained unclear. It needs to be elucidated in the future studies, whether *P. rubens* is actually capable of hydrolyzing lovastatin or if the conditions of submerged co-cultivation promote the observable “leakage” of this precursor metabolite from the mycelium of *A. terreus*. Finally, it should be mentioned that the enzymatic hydrolysis of lovastatin can be applied to generate the semi-synthetic statin drugs (Schimmel et al. 1997). In this context, further verification of *C. globosum* abilities to degrade lovastatin, possibly via the esterase-mediated catalytic mechanisms, would be required.

**Fig. 7:**
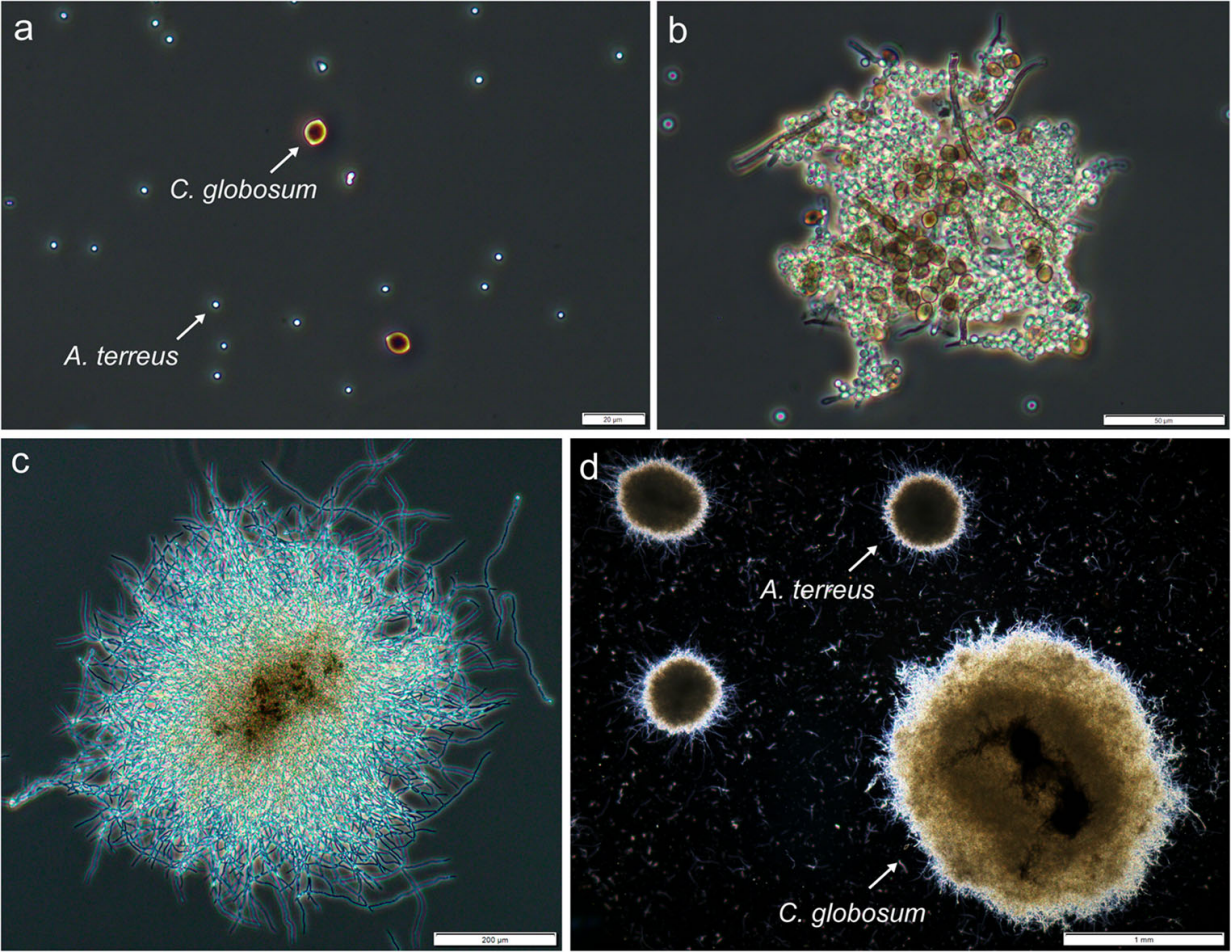
Microscopic images corresponding to *A. terreus* and *C. globosum* co-cultures. The photographs of fungal spores at the moment of inoculation (**a**), the agglomerates formed after 8 h of co-cultivation (**b**), and the pellet developed in co-culture after 24 h (**c**) represent the “spores/spores” approach of co-culture initiation. The pellets shown in **d** were observed at the end of the co-culture which was started by combining the 24-h mycelia of *A. terreus* and *C. globosum*. The scale bars represent 20 μm, 50 μm, 200 μm, and 1 mm in **a**, **b**, **c**, and **d**, respectively

Since the spores of *A. terreus* and *C. globosum* differ markedly in the appearance and can be distinguished with the use of a standard light microscope (Fig. 7a), it was possible to observe the co-agglomeration of spores taking place in the co-culture (Fig. 7b). The agglomerates then gave rise to pellets which had the spores of both species embedded in their cores, with lemon-shaped, larger spores of *C. globosum* tightly packed with smaller spores of *A. terreus* (Fig. 7c). If the pellets developed in 24-h or 72-h monocultures were contacted instead of spores, the evident differences in size were typically observed, meaning the *C. globosum* pellets, containing the clearly visible fragments of black fruiting bodies in their cores, were typically larger than the pellets of *A. terreus* (Fig. 7d). In terms of metabolites production, confronting the spores (Fig. 2d) or the 72-h cultures (Fig. 4d) of *A. terreus* and *C. globosum* led to the elevated levels of butyrolactone I compared with the tested standard monocultures. Interestingly, butyrolactone I was previously suggested to act as a signaling molecule involved in quorum sensing (Palonen et al. 2014). The characteristics of *C. globosum* responsible for such behavior were unknown; however, the co-agglomeration of spores was not shown to be an essential condition in this respect, as in the “72-h mycelium/72-h mycelium” experiments, the well-developed, mature pellets were confronted instead of spores and the stimulating effect was still observable.

If the results of “spores/spores” confrontation between two (Fig. 2) and four (Fig. 5) species are considered, it appears the extensive load of foreign spores in the four-species variant could have inhibited the growth of *A. terreus* and thus led to relatively low metabolite levels. In fact, in the two-species runs, there was an approximately equal number of spores corresponding to the two involved species, whereas in the four-species experiment, the spores of *A. terreus* at the concentration of 10^9^ per liter were contacted with 10^9^ spores per liter of each of the three accompanying species. Therefore, it turned out that *A. terreus* did not perform well in this particular setup, despite the favorable medium composition. On the other hand, when the fully developed pellets were used to initiate the four-species co-culture instead of spores, the levels of secondary metabolites produced by *A. terreus* were relatively higher, as the early developmental stages of the fungus were not affected (Fig. 5).

The co-cultures initiated by introducing the spores of *A. terreus* into the 24-h monocultures of *P. rubens* and *C. globosum* resulted in the relatively low levels of secondary metabolites (Fig. 3). In the case of *A. terreus*/*M. racemosus*, however, the recorded levels of lovastatin, (+)-geodin, and asterric acid were visibly higher (Fig. 3). This behavior was probably associated with the morphological characteristics of the tested strains. In short, *P. rubens* and *C. globosum* are known to exhibit the agglomerative mechanism of submerged development, whereas *M. racemosus* is a non-agglomerative species (Kowalska et al. 2018). It is likely that the biomass of *P. rubens* and *C. globosum* interfered with the agglomeration of *A. terreus* spores, possibly by “sweeping” or “trapping” the spores into the already developed mycelial aggregates or pellets. In other words, the spore-based inoculum of *A. terreus* was negatively affected by the foreign fungal biomass of agglomerative nature. This detrimental effect was less visible in the presence of *M. racemosus*, because the non-agglomerated hyphae of this species were less likely to hinder the formation of aggregates commonly observed for *A. terreus* cultures under submerged conditions.

If one compares the outcomes of submerged mono- and co-cultivation runs performed in the present study, it is clear that the co-cultivation approach led either to the stimulation or inhibition of biosynthetic routes in *A. terreus* depending on the strategy of co-culture initiation and the choice of the co-cultivation partner. It is important to discuss the factors contributing to the observed mono-/co-culture differences. Firstly, as the co-cultivation proceeds, the two (or more) strains become involved in the chemical interactions due to the myriad of molecules biosynthesized and secreted into the liquid medium by each of them. This was particularly evident when the 72-h monocultures were combined to trigger the co-cultivation, as in this case, one of the fungal partners was already in the “mature” phase of the monoculture and a wide spectrum of fungal metabolites and proteins was expected to be already present in the medium at this time. The impact of such strain-specific “molecular cocktails” on other strains is yet impossible to be predicted and fully understood. Even though the consequences of the interactions can be monitored in a chosen context, as it was done in the present study with regard to the production of secondary metabolites, the underlying molecular events and mechanisms still remain to be examined and elucidated. The complexity of the problem is also related to the possibility of the chemical modifications that may take place whenever an enzyme produced by one species displays affinity towards the metabolites produced by the other species. Secondly, if two or more fungal strains co-exist in the culture, they are expected to compete for nutrients necessary for their survival. Consequently, since the biosynthesis of secondary metabolites, including lovastatin, is typically regarded as responsive with respect to the availability of the sources of carbon, nitrogen, and other elements, the formation of metabolic products in the co-culture would always be somehow associated with the relative rates of substrates consumption exhibited by the respective strains. For example, if two fungal strains utilize lactose or yeast extract components at markedly distinct rates, their relative impact on the biosynthetic capabilities of *A. terreus* will also be different. In other words, the differences with regard to substrates availability in mono- and co-cultures are expected to be reflected by the concentration of secondary metabolites in the broth. Importantly, the supply of oxygen should also be taken into consideration here. Thirdly, the inter-species confrontation may affect the development and morphological evolution of the involved fungi, e.g., when the spores or pellets of one species interfere with the agglomeration and germination of other species’ spores. This situation was observed in the case of “spores/24-h mycelium” experiment, where the fungal spores added to the culture at 24 h were at the danger of being “swept” by the already developed pellets representing the other species. This strategy could be regarded as deliberately putting one of the species at a developmental disadvantage. In addition to the factors described above, one should also consider the mechanical and bioprocess-related consequences of multi-species cultivation, i.e., the characteristics of the broth, e.g., viscosity, and the mechanical interactions between dispersed hyphae, pellets, and clump forms. The picture gets even more complicated if the correlation between the morphological forms, broth viscosity, and oxygen transfer is accounted for. Finally, one cannot exclude the possibility of inter-species interactions via the molecules expressed on cell surfaces. Altogether, there are many aspects of submerged co-cultivation a variety of research topics that deserve further exploration and may be addressed in the future.

To conclude, the present study has shown that the outcomes of submerged co-cultivation of *A. terreus* with other filamentous fungi clearly depend both on the strategy of co-culture initiation and the choice of co-cultivated species. Even though the suggested strategy does not seem to be effective in terms of enhancing lovastatin production compared with the conventional monocultures, it considerably affects the biosynthetic performance of *A. terreus* and can be regarded as promising with respect to elevating or decreasing the levels of butyrolactone I, asterric acid, and (+)-geodin. Furthermore, the presence of *C. globosum* as the co-culture partner of *A. terreus* leads to the relatively high amounts of a molecule that can be potentially applied as a substrate for the synthesis of statin drugs. Finally, the study revealed the co-agglomeration of *A. terreus* and *C. globosum* spores under the conditions of submerged co-cultivation.

## Electronic supplementary material

ESM 1 (PDF 362 kb)
