# Supplementary material for: Evaluating the outcomes of submerged co-cultivation: production of lovastatin and other secondary metabolites by Aspergillus terreus in fungal co-cultures
Source: Appl Microbiol Biotechnol. 2019 May 16;103(14):5593–605. doi: 10.1007/s00253-019-09874-0 (PMC6597594; doi:10.1007/s00253-019-09874-0)
Supplement: Supplementary file 1 — (PDF 362 kb) [file 253_2019_9874_MOESM1_ESM.pdf]

Applied Microbiology and Biotechnology

Supplementary material

"Evaluating the outcomes of submerged co-cultivation: Production of lovastatin and other secondary metabolites by *Aspergillus terreus* in fungal co-cultures"

Tomasz Boruta*, Iwona Milczarek, Marcin Bizukojc

Lodz University of Technology, Faculty of Process and Environmental Engineering, Department of Bioprocess Engineering, ul. Wolczanska 213, 90-924 Lodz, Poland

* Corresponding author. Phone: +48 42 631 39 77; fax: +48 42 636 56 63

E-mail address: tomasz.boruta@p.lodz.pl

The supplementary material contains 1 figure.

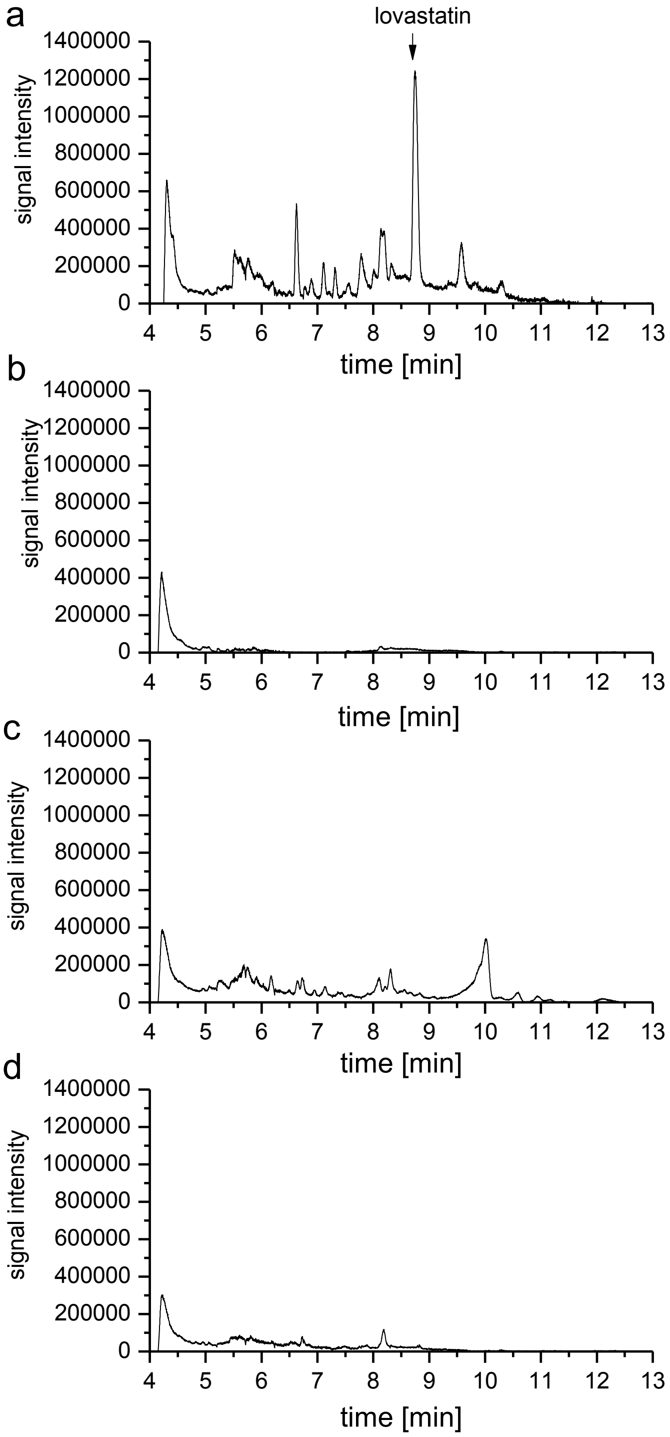

Fig. S1 Total ion chromatograms of monoculture controls of *A. terreus* (a), *M. racemosus* (b), *P. rubens* (c) and *C. globosum* (d)
